# The anti-*Toxoplasma* activity of the plant natural phenolic compound piceatannol

**DOI:** 10.3389/fvets.2022.972500

**Published:** 2022-08-02

**Authors:** Yucong Jiang, Yuehong Shi, Dandan Hu, Xingju Song

**Affiliations:** College of Animal Science and Technology, Guangxi University, Nanning, China

**Keywords:** *Toxoplasma gondii*, natural extraction products, piceatannol, antiparasitic, drug discovery

## Abstract

*Toxoplasma gondii* is an obligate intracellular protozoan that infects the nucleated cells of warm-blooded animals and causes life-threatening disease in immunocompromised patients. Due to the limited effectiveness and prominent side effects of existing drugs, there is an urgent need to develop new therapeutic options against *T. gondii*. Piceatannol is a natural plant compound with multiple functions such as antibacterial, antileukemic and antiparasitic activities. In the present study, the anti-*T. gondii* activity of piceatannol was evaluated. Piceatannol potently inhibited *Toxoplasma* with a half-maximal effective concentration (EC_50_) of 28.10 μM. Piceatannol showed a significant inhibitory effect on intracellular proliferation, inhibiting intracellular parasites at a rate of 98.9% when treatment with 100 μM piceatannol. However, the invasion ability of tachyzoites was not affected by piceatannol. By immunofluorescence assay, we noted that the parasite showed abnormalities in cell division after exposure to piceatannol. To determine the *in vivo* effect of piceatannol on acute infection, a model was established by infecting BALB/c mice with the virulent RH strain of *T. gondii*. Mice infected with 500 tachyzoites showed a significant therapeutic effect when treated with 15 mg/kg of piceatannol. These results suggest that piceatannol is a promising drug for the treatment of *T. gondii*.

## Introduction

*Toxoplasma gondii* is a zoonotic parasite that infects almost all warm-blooded animals and humans worldwide. The infection is usually asymptomatic in immunocompetent individuals. However, serious consequences may occur in immunocompromised individuals (1). In patients with HIV or receiving chemotherapy for cancer or organ transplants, active *T. gondii* infections mainly lead to encephalitis, pneumonia or chorioretinitis, and tissue destruction in other organs (2, 3). Women with major infections during pregnancy may result in fetal death, malformation or miscarriage. The main route of infection in humans is through the ingestion of meat containing cysts or food contaminated with oocysts. In addition, people may become infected through blood transfusions, organ transplants and transplacental transmission (4). *T. gondii* infections have caused serious public health problems. One-third of the world's population is estimated to be infected, and severe outbreaks of toxoplasmosis have occurred in several countries (5, 6).

Currently, clinical treatment of toxoplasmosis relies on chemical drugs. A combination of sulfadiazine and pyrimethamine has become the standard therapy for toxoplasmosis (7). However, this combination still has a high failure rate and is ineffective against chronic infections. Moreover, different severe complications such as teratogenic potential, reversible myelosuppression, neutropenia, thrombocytopenia, hypersensitivity reactions and hepatic necrosis have been reported (8–11). Furthermore, other drugs such as azithromycin, clarithromycin, spiramycin, atovaquone and cotrimoxazole (trimethoprim-sulfamethoxazole) are also commonly used in clinical toxoplasmosis. However, these drugs are less effective than conventional treatment and are often accompanied by severe side effects and incomplete treatment (12–14). For example, spiramycin treatment alone could significant reduction in mother-to-child transmission (MTCT) rates of diagnosed maternal *T. gondii* infection, but if fetal infection is suspected or confirmed pyrimethamine–sulfonamide–folinic acid should be used (15). Thus, the limitations of available treatment options underscore the urgent need for better treatment options for acute and latent toxoplasmosis.

Recently, natural products have been considered as good alternatives for the development of *T. gondii* drugs (16). Various studies have shown that natural extracts/components of plants have inhibitory effects on *T. gondii*. Compounds from antimalarial plants, such as *Artemisia annua, Cinnamomum comphora, Lippia multiflora* and *Vernonia colorata*, were found to be effective against *T. gondii* (17). Some other plant extracts such as essential oils from *Lavandula angustifolia* and *Pelargonium X*, Vernodalin from *V. colorata*, TAF355 and TAF401 from *Eurycoma longifolia*, and Ginkgolic acids from *Ginkgo biloba* also have a good inhibitory effect on *T. gondi*i (18–21). Resveratrol, a polyphenol family of stilbene molecules from plants, has potent inhibitory activity against several important protozoa, including *Toxoplasma, Leishmania*, and *Amoeba* (22, 23). Piceatannol is a natural analog of resveratrol and is mainly found in passion fruit (*Passiflora edulis* Sims), blueberries, grapes, sugarcane, white tea, and rhubarb (24–26). Piceatannol has been reported to possess antioxidant, anti-proliferative, immune enhancement, anti-inflammatory, anti-thrombotic, anti-cancer, anti-hyperlipidemic and antibacterial activities and is widely used for the prevention/treatment of heart disease, leukemia, cancer, etc. (27). However, it remains unclear whether piceatannol has anti-*T. gondii* effects and the mechanism behind the clearance of intracellular parasites.

In this study, we evaluated the inhibitory effect of piceatannol against *T. gondii*. Our results indicate that piceatannol can potently inhibit *T. gondii* with a high *in vitro* safety index. These results suggest that piceatannol is a promising drug candidate for the treatment of toxoplasmosis.

## Results

### Piceatannol has a potent anti-*Toxoplasma* activity

**Figure 1:**
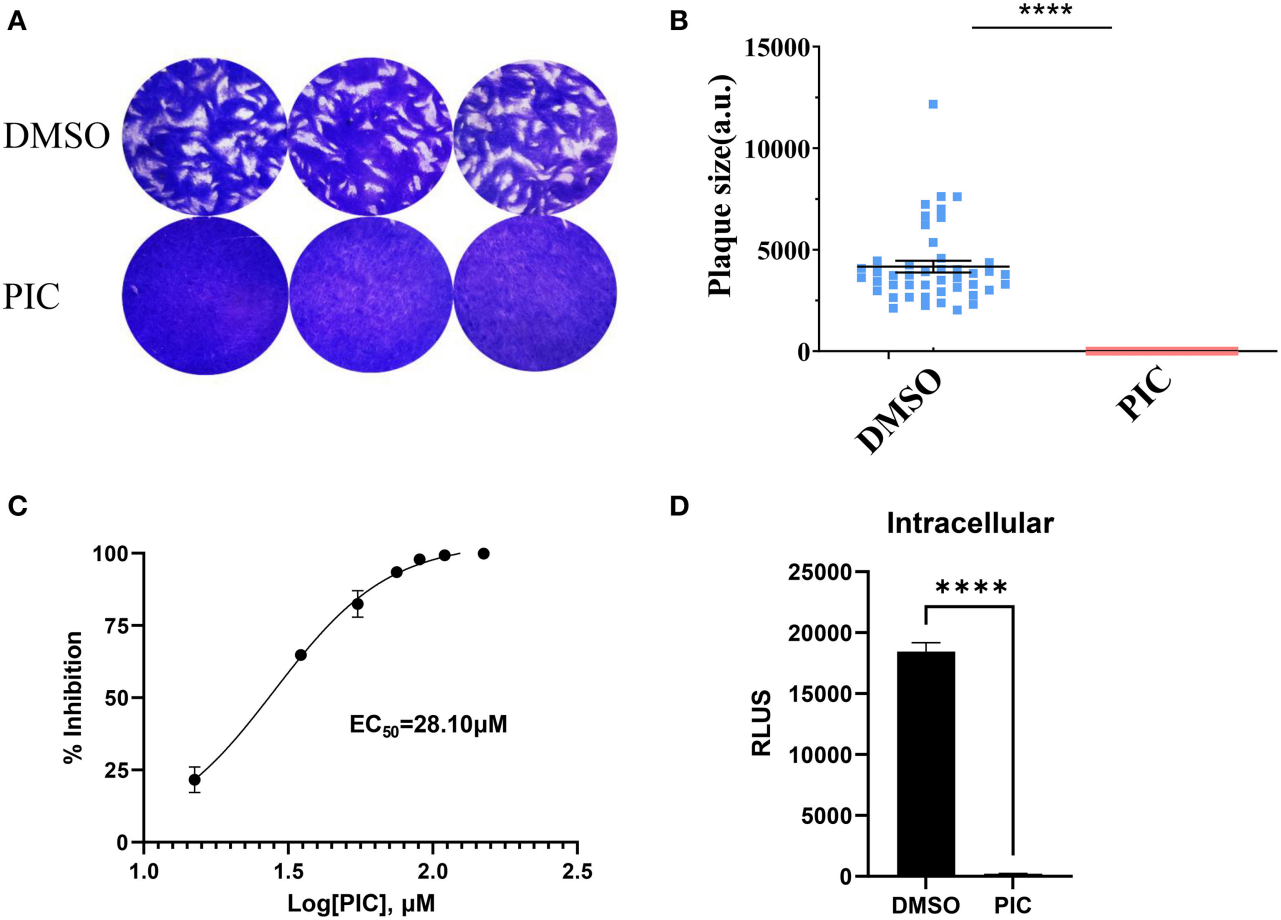
Piceatannol displaying a dose-dependent inhibitory effect against TgRH-Luc tachyzoites. **(A)** The Plaque assay for comparing the overall proliferation ability of the parasites after piceatannol and DMSO treatment. TgRH-Luc tachyzoites were added to HFF cells cultured in 12-well plates, with 150 tachyzoites added to each well. Infected cells were treated with DMSO and 100 μM Piceatannol, respectively. Cells were cultured for 7 days followed by fixation with PFA and staining with 0.2% crystal violet. **(B)** Measurement of plaque areas using the pixel point using Pixel in the Photoshop C6S software (Adobe, USA). Data were obtained from three independent experiments. Statistical analysis of the plaque area was performed by Graph Pad Prism using Student's *t* test. Asterisks indicate *p* < 0.0001. **(C)** Inhibition curve of piceatannol on *T. gondii in vitro*. TgRH-Luc was treated with different concentrations of piceatannol ranging from 0 to 150 μM, and relative RLU was detected after 24 h. Percentage proliferation inhibition was calculated as: inhibition rate = [(RLU_DMSO_ - RLU_treatment_)/RLU_DMSO_] × 100. EC_50_ was calculated using the log (inhibitor) vs. response-variable slope (four parameters) regression equation. The results are shown as the mean ±SEM from three independent experiments. **(D)** Piceatannol inhibition of intracellular *T. gondii*. Freshly released tachyzoites (1 × 10^5^) were allowed to invade the host cells for 3 h at 37° C and 5% CO_2_, and then DMEM medium containing piceatannol or DMSO was added after wash three times. The infected cells were cultured for 24 h until fluorescence was detected. Data were obtained from three independent experiments. Statistical analysis of the plaque area was performed by Graph Pad Prism using Student's *t* test. Asterisks indicate *p* < 0.0001.

The plaque assay was used to comprehensively evaluate the proliferation of RH *T. gondii* tachyzoites treated with piceatannol and DMSO during the entire lytic cycle. The results showed no plaque formation after piceatannol treatment, which differed significantly from the DMSO control group (Figures 1A,B). To determine the effect of different concentrations of piceatannol on tachyzoites, the RH strain of *T. gondii* expressing luciferase (TgRH-Luc) was used, as indicated by the reduced luciferase activity. The parasites in African green monkey kidney (Vero) cells were determined at different piceatannol concentrations using an *in vitro* drug inhibition assay. The results showed a good inhibitory effect of piceatannol on TgRH-Luc in a dose-dependent manner (Figure 1C). The half-maximal effective concentration (EC_50_) of piceatannol was recorded to be 28.10 μM (95% confidence interval [CI], 25–30 μM). Since piceatannol has an inhibitory effect on the development of *T. gondii*, it is necessary to further investigate whether it acts on intracellular tachyzoites. Intracellular parasites were treated with 100 μM piceatannol. The inhibition rate was 98.9% for the intracellular parasites (Figure 1D). The results indicate that piceatannol has good inhibitory effect on intracellular *T. gondii*.

### Inhibition of intracellular proliferation of *T. gondii* by piceatannol

**Figure 2:**
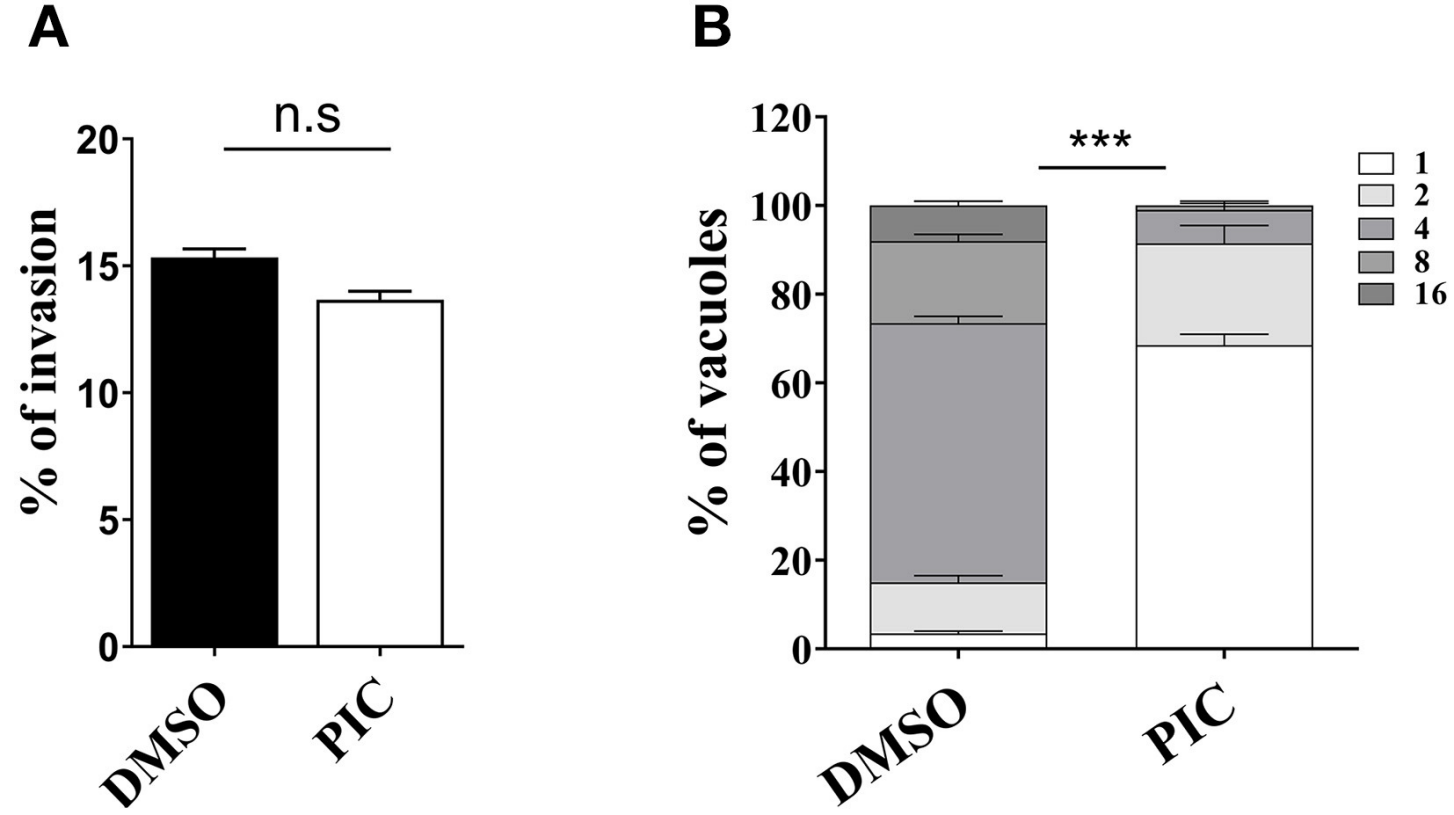
Effect of piceatannol on parasite invasion and proliferation. **(A)** Graph of RH invasion suppression efficiency. RH infection was cultured in Vero cells containing 100 μM piceatannol or DMEM and IFA was performed to calculate the rate of RH invasion. Statistical analysis of the plaque area was performed by Graph Pad Prism using Student's *t* test. **(B)** Graph of RH proliferation inhibition efficiency. After infecting Vero cells with RH for 3 h, infected cells were treated with 100 μM piceatannol. The proportion of vacuoles containing 2, 4, 8 and 16 tachyzoites of the RH strain was calculated under fluorescence microscopy and the average value was taken three times. Statistical analysis of the plaque area was performed by GraphPad Prism using two-way ANOVA. n.s, non-significant, ***, *p* < 0.001.

*T. gondii* tachyzoites in cells involves a complete set of lytic cycles, including invasion, intracellular replication and egress (28). The reduction in plaque formation may be caused by impairment of one or more steps of the lytic cycle. Thus, we next sought to investigate which lytic cycle of *T. gondii* affects piceatannol. We primarily assessed parasite invasion processes, which showed no significant differences between the piceatannol and DMSO treatment groups (Figure 2A). Then, intracellular proliferation was assessed by observing the number of tachyzoites in the vacuoles after piceatannol and DMSO treatment. The results showed that piceatannol potently inhibited the intracellular proliferation of *T. gondii* (*p* < 0.001) (Figure 2B). These results suggest that the reduction in plaque size induced by piceatannol treatment is specifically due to the impairment of intracellular proliferation of the parasites.

### Treatment with piceatannol reduces parasite virulence in mice

**Figure 3:**
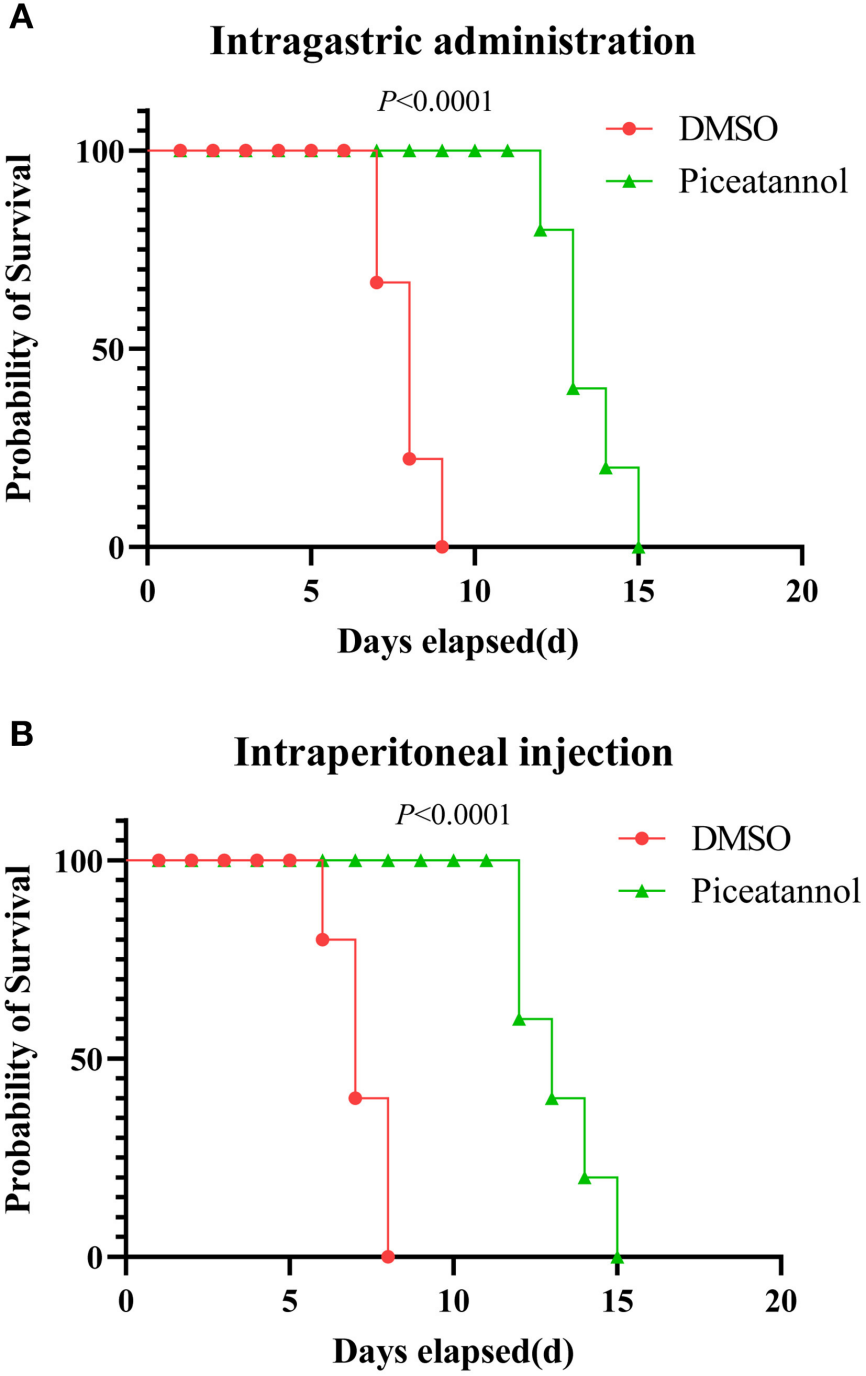
Therapeutic effect of piceatannol on *T. gondii* infection in mice. BALB/c mice were infected with 500 tachyzoites by intraperitoneal injection. Then, mice were treated with 15 mg/kg piceatannol or DMSO by intragastric administration **(A)** or intraperitoneal injection **(B)**. The results show a therapeutic effect of piceatannol on mice infected with parasites. Statistical analysis was performed with survival curve of Graph Pad Prism using Log-rank (Mantel-Cox) test (San Diego, CA).

We further examined the effect of piceatannol on parasite proliferation *in vivo*. Two delivery routes (intragastric administration and intraperitoneal injection) were employed independently. In the intragastric administration experiment, 40% of the mice in the DMSO group died on day 7 and the remaining 60% died on day 8 and 9, whereas the death of the mice in the piceatannol-treated group was significantly delayed (*p* < 0.0001), with the last one dying on 15 days post infection (Figure 3A). In the intraperitoneal injection experiment, all mice in the DMSO group died by day 8, whereas death was significantly delayed in the piceatannol-treated group (the last mouse died 15 days after infection) (Figure 3B). These results suggest that piceatannol inhibits the proliferation of *T. gondii in vitro*; however, it does not completely kill the parasite in the mice.

### Piceatannol exposure causes abnormal division

**Figure 4:**
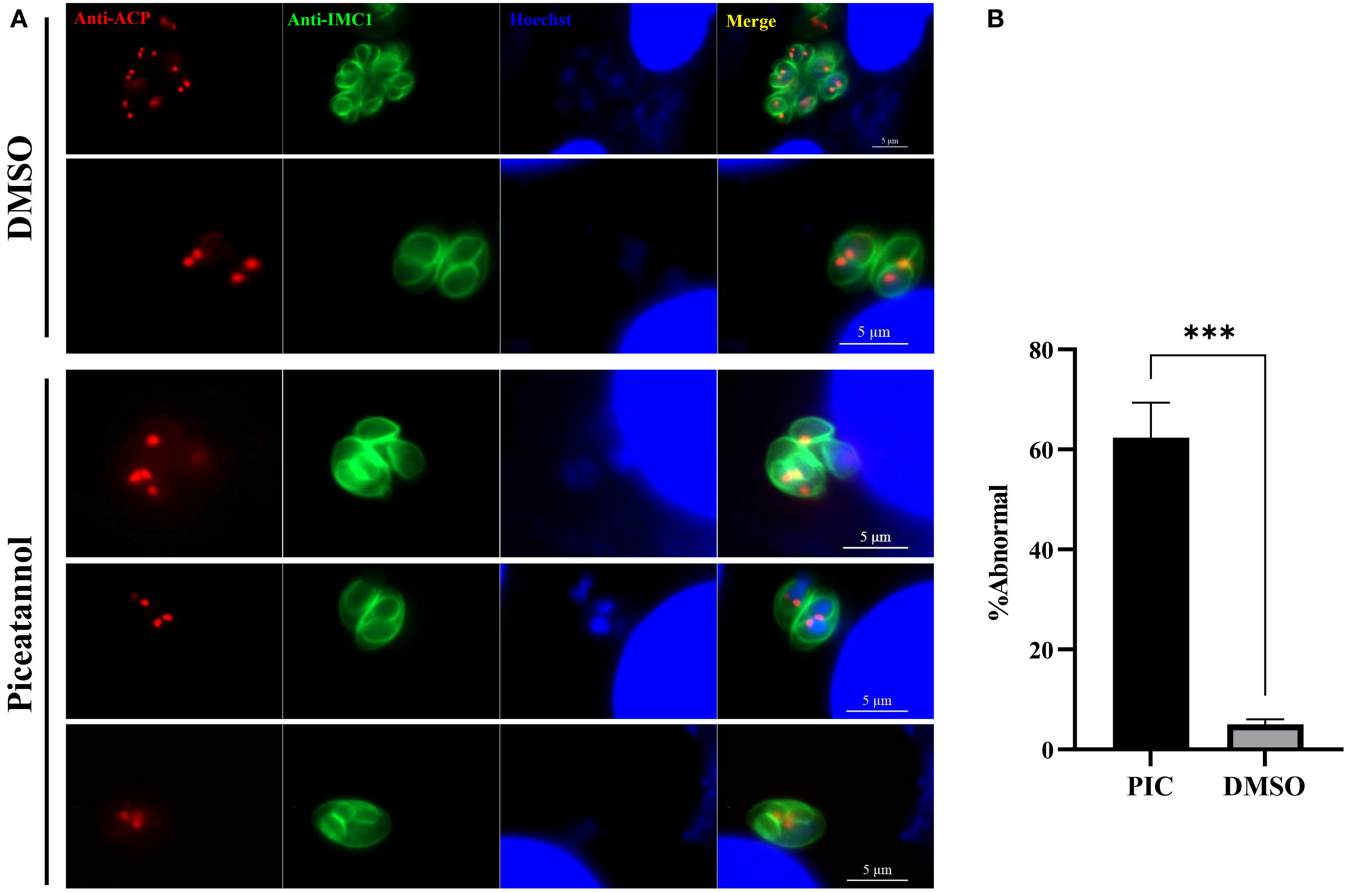
Piceatannol exposure resulting in abnormal development of *T. gondii*. Indirect immunofluorescent assay was performed after 100 μM piceatannol treatment to observe the dividing of the parasite. Organelles including IMC, apicoplast and nuclei were stained by using rabbit anti-IMC1 antibody, mouse anti-ACP antibody and Hoechst dye, respectively. **(A)** Abnormal division of *T. gondii* after piceatannol or DMSO treatment. **(B)** The proportion of abnormal divided parasites after piceatannol or DMSO treatment. The proportion of vacuoles containing abnormally splitting tachyzoites was calculated under fluorescence microscopy and the average value was taken three times. Statistical analysis of the plaque area was performed by Graph Pad Prism using Student's *t* test. ***, *p* < 0.001.

Considering the inhibitory effect of piceatannol on parasite proliferation, we sought to determine whether treatment with piceatannol would alter the division of tachyzoites. Immunofluorescence assays were performed to observe the morphology of the parasites after treatment. We noticed that piceatannol treated tachyzoites in a single PV divided asynchronously, and even three daughter cells were found in a single tachyzoite. The number of tachyzoites in untreated *T. gondii* vacuoles was 2, 4, 8, 16, etc. due to their binary fission pattern. However, a large number of abnormal divisions were observed after piceatannol treatment (Figure 4A). We found an odd number of parasites (e.g., 3 or 5 tachyzoites) predominantly present in a single vacuole (Figure 4A). We found ~60% tachyzoites showed abnormal division after piceatannol treatment, while only 5% were found in DMSO treated group (Figure 4B). These results suggested that piceatannol inhibit parasite proliferation by suppressing its cell division.

### Piceatannol is not toxic to host cells at antiparasitic concentrations

**Figure 5:**
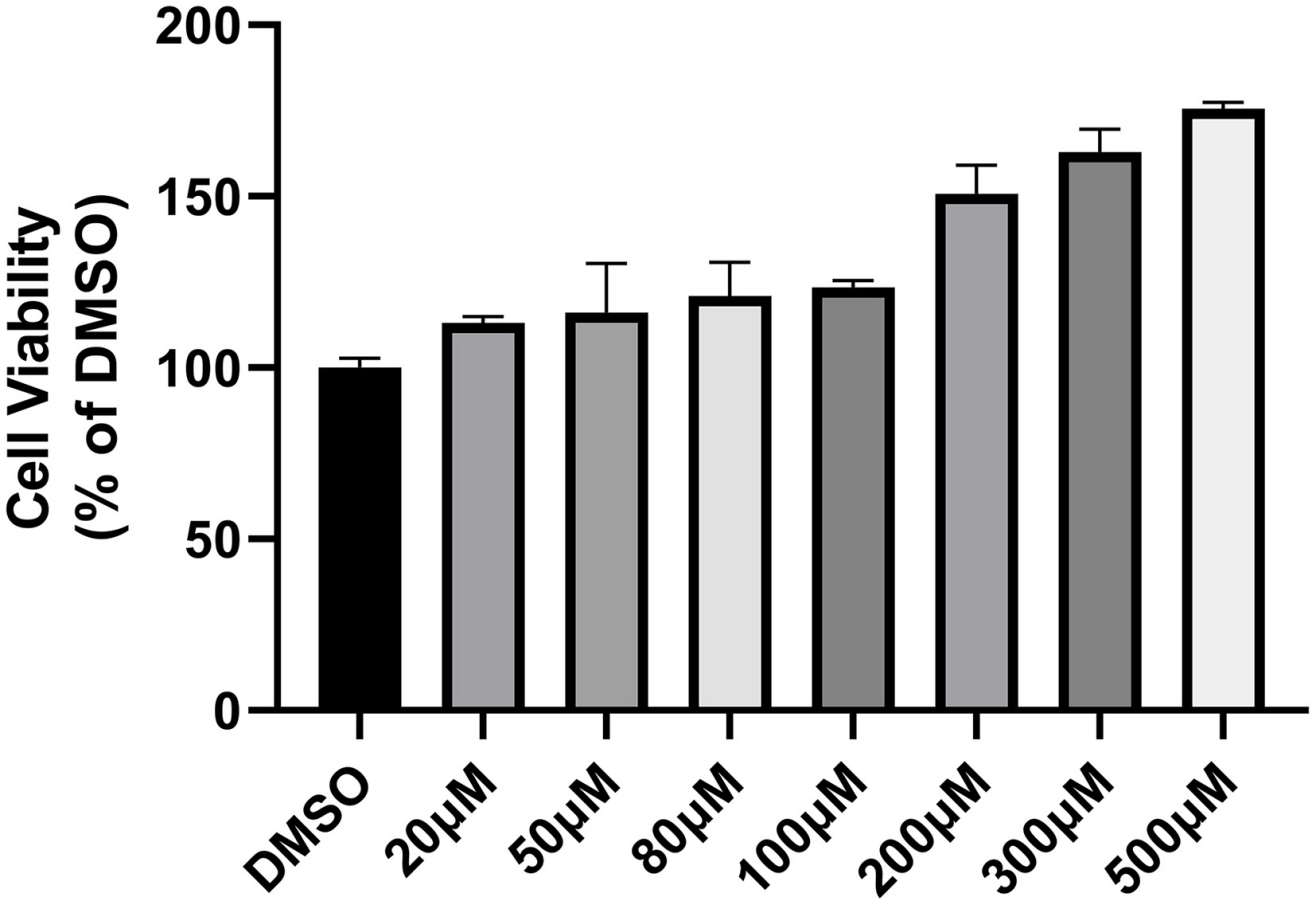
Cytotoxicity of piceatannol on Vero cells. Cytotoxicity assay graph. Toxicity of 20–500 μM piceatannol on Vero cells was measured using CCK-8 reagent. DMSO was set as a control. Inhibition rate = (OD _DMSO_-OD _PIC_)/OD _DMSO_. Piceatannol was not toxic to Vero cells. This experiment was performed in triplicate.

To assess the cytotoxic effect of piceatannol on host cells, sequentially diluted piceatannol from 20 μM to 500 μM was added to host cells and cell viability was determined using CCK-8 reagent. The results showed that in the therapeutic concentration range, piceatannol was not toxic to cells and that high concentrations of piceatannol had a promotive effect on the proliferation of Vero cells (Figure 5).

## Discussion

Toxoplasmosis, as a zoonotic disease, has serious public health implications worldwide and also poses serious hazards to humans and domestic animals. Although most human infections have no complications, they can still be fatal or cause serious problems in fetuses and immunocompromised patients (5). Currently, clinical treatment of toxoplasmosis still relies on chemical drugs. However, this treatment is often accompanied by severe side effects (8–11). Recently, a large number of studies have focused on the use of natural plant extracts for the treatment of toxoplasmosis, which offers the possibility of developing anti-*T. gondii* drugs with high activity, high efficiency, low toxicity, and low cost. Artemisinin and its derivatives have long been discovered and characterized as potent antimalarials, which have revolutionized the treatment of malaria and are being actively studied in *T. gondii* (29, 30). However, drug resistance has limited their widespread use (31–33). Extracts from some antimalarial plants, such as *Glycyrrhiza glabra*, have anti-proliferative effects against *T. gondii* by directly inhibiting the parasite in the cells of infected hosts (34). In addition, the essential oils extracted from *Lavandula angustifolia* and *Pelargonium X. asperum*, the TAF355 and TAF401 from *Eurycoma longifolia* also showed good inhibitory effect on *T. gondii* (19–21). Therefore, the search for anti-*T. gondii* natural products is very promising.

Resveratrol is a natural polyphenolic compound, which can reduce the number of extracellularly grown tachyzoites, probably by disrupting the redox homeostasis of the parasites. Moreover, resveratrol is capable of releasing the burden of cellular stress and promoting apoptosis in *T. gondii* (26). Piceatannol is a natural analog of resveratrol, which found mainly in passion fruit, blueberry, grape, sugarcane, white tea, rhubarb, etc. (35). It has various biological activities such as antioxidant, anti-proliferative, immune enhancement, anti-inflammatory, anti-thrombotic, anti-cancer, anti-hyperlipidemic, and antibacterial (27). In this study, we characterized the anti-*T. gondii* properties of piceatannol. Our results suggest that piceatannol effectively inhibits *T. gondii* intracellularly and extracellularly, with an EC_50_ of 28.10 μM on RH tachyzoites.

In this study, we characterized the anti-*T. gondii* properties of piceatannol. Our results suggest that piceatannol effectively inhibits *T. gondii* intracellularly with an EC_50_ of 28.10 μM on RH tachyzoites. The proliferation of *T. gondii* tachyzoites in cells involves a complete set of lytic cycles, including invasion, intracellular replication and egress (28). To investigate which lytic cycle is affected by piceatannol, we performed invasion and proliferation experiments. The invasion rate of tachyzoites did not change significantly after piceatannol treatment, while proliferation was greatly affected. Thus, it is hypothesized that piceatannol reduces the number of plaques in the plaque assay by inhibiting the proliferation of tachyzoites in the cells. Piceatannol has various biological activities such as antioxidant, anti-proliferative, immune enhancement, anti-inflammatory, anti-thrombotic, anti-cancer, anti-hyperlipidemic, and antibacterial (27). It is a natural analog of resveratrol, which found mainly in passion fruit, blueberry, grape, sugarcane, white tea, rhubarb, etc. (35). Previous research showed that resveratrol can reduce the proliferation of tachyzoites, probably by disrupting the redox homeostasis of the parasites. Moreover, resveratrol is capable of releasing the burden of cellular stress and promoting apoptosis in *T. gondii* (26). Thus, we hypothesize that the mechanism of piceatannol inhibits *T. gondii* development when before and during cell infection might same as resveratrol.

Further studies showed that treatment with piceatannol resulted in abnormal division of parasites. Since *T. gondii*, as a single-celled eukaryote, undergoes binary division, tachyzoites in parasitophorous vacuoles are usually divided synchronously, the number of tachyzoites in a PV is multiples of 2 (36). Unusually, the piceatannol-treated group showed a large number of abnormal divisions, e. g., asynchronously division and even three daughter cells emerged in a single tachyzoite and 3 or 5 tachyzoites per vacuole were presented. These results suggest that piceatannol may affect *Toxoplasma* division, which may be one of the reasons why piceatannol affects the proliferation of parasites.

Usually, such anti-*Toxoplasma* drugs are toxic to cells. However, in the present study, we found that piceatannol had no toxic effect on cells and it even promoted cell proliferation at higher concentrations. Previous studies have shown that piceatannol enhances the viability of HG-induced H9C2 cardiac myoblasts (37). However, the mechanism of how piceatannol improves cellular activity is unclear and needs to be determined by further studies.

Potent *in vitro* parasite killing and non-toxicity make piceatannol a good anti-*Toxoplasma* candidate. However, piceatannol was not significantly effective in the treatment of mice infected with *T. gondii*. Although it had some effect in delaying the death of mice, it did not prevent the death of mice. We speculate that this may be due to the rapid metabolism in mice. A previous study showed that the blood concentration of piceatannol peaked at 15 min after intragastric administration and decreased significantly after 2 h. Piceatannol was totally metabolized after 4 h of treatment, and a large amount of piceatannol metabolites were found in the urine of rats (38). Therefore, the structure of piceatannol can be further optimized to delay its metabolism *in vivo* and thus improve its therapeutic effect in *T. gondii-infected* mice.

## Conclusion

In this study, we characterized the anti-*T. gondii* properties of piceatannol. Piceatannol affected the division and morphology of *T. gondii* and exhibited potent anti-*Toxoplasma* effects *in vitro* without cell toxicity. Our study suggests that piceatannol is a promising drug for the treatment of *T. gondii*.

## Materials and methods

### Ethics statement

Animal experiments were conducted in strict accordance with the recommendations of the Guide for the Care and Use of Laboratory Animals of the Ministry of Science and Technology of China. All experimental procedures were approved by the Institutional Animal Care and Use Committee of Guangxi University.

### Parasites, drugs and cell culture

Human foreskin fibroblasts (HFFs) and green monkey kidney (Vero) cells were purchased from the American Type Culture Collection (Manassas, VA, United States) and cultured in Dulbecco's Modified Eagle Medium (DMEM) supplemented with 10% fetal bovine serum (FBS). In this study, a type I RH strain of *T. gondii* constitutively expressing firefly luciferase (TgRH-Luc) at the UPRT site was used and transferred to Vero cells. Parasites were cultured in DMEM supplemented with 2% fetal bovine serum. The drug monomer piceatannol was purchased from Aladdin Chemical Reagent Company (Aladdin, Shanghai, China).

### *In vitro* inhibition assay

Vero cells were seeded onto 96-well cell plates and cultured at 37°C with 5% CO_2_. Cells were then infected with 1 × 10^5^ of TgRH-Luc per well. At 24 h post infection (h.p.i.), 2-fold serial dilutions of piceatannol (final concentration from 57 to 0.22 μM) were added to each parasite-infected well. Equal amounts of DMSO were used as controls. Relative luminescence units (RLU) were detected after 24 h of treatment by a fluorescence microplate reader (Tecan, Infinite M200 PRO, Männedorf, Switzerland) using the Bright-Lumi™ II Firefly Luciferase Assay Kit (Beyotime Biotech, Shanghai, China). Percentage proliferation inhibition was calculated as: inhibition rate = [(RLU_DMSO_ - RLU_treatment_)/RLU_DMSO_] × 100%. Samples were run in triplicate and three independent assays were performed. Data are expressed as mean ± standard deviation (SD). Half-maximal effective concentration (EC_50_) of compounds and 95% confidence interval (CI) were extrapolated using the log (inhibitor) vs. response-variable slope (four parameters) regression equation in GraphPad Prism 8 (GraphPad, La Jolla, CA).

### Plaque assays

In order to make a preliminary identification of the anti-*T. gondii* ability of piceatannol, plaque assays were performed as described previously (39). Briefly, purified parasites were used to infect HFF seeded on 12-well-plates (150 tachyzoites/well). HFF cells were treated with 100 μM Piceatannol and DMSO-treated cells were used as a control. After 7 days of undisturbed culture, HFF were fixed with 4% PFA and stained with crystal violet. Cells in the plaque area were counted in pixels using Photoshop C6S software (Adobe, USA), and data from three independent experiments were compiled.

### Intracellular inhibition assays

Vero cells growing in 96-well-plates were infected with 1 × 10^5^ TgRH-Luc parasites for 3 h and then cultured with piceatannol (100 μM) for 24 h at 37°C with 5% CO_2_. Cells and parasites were then lysed, and RLUs were measured as described above.

### Intracellular replication assay and invasion assay

An intracellular replication assay was performed to assess the number of parasites per vacuole 24 h after invasion, which is consistent with a previous description (40). Briefly, HFF growing in 12-well-plates seeded on coverslips were inoculated with 1 × 10^5^ parasites and cultured continuously for 2 h. They were subsequently treated with 100 μM piceatannol for 24 h, and DMSO-treated cells were used as a control. Then, an indirect immunofluorescent assay was performed using rabbit anti-GAP45 antibodies and Hoechst dye to observe the intracellular replication of tachyzoites. The tachyzoites of each strain in the vacuoles were quantified by counting at least 100 vacuoles using a fluorescence microscope (Zeiss, Germany). For the invasion assay, the percentage of invasion was calculated based on the number of vacuoles per host cell. Three independent experiments were performed.

### Effect of piceatannol on *T. gondii* infection in mice

Six-week-old BALB/c mice were infected with 500 tachyzoites by intraperitoneal injection. The parasite-infected mice were divided into four groups of five mice each. After 1 day infection, the mice were treated with 15 mg/kg/d of piceatannol by intragastric administration (group A) and intraperitoneal injection (group B) for 15 days. DMSO was treated as a control group accordingly. Survival was evaluated for up to 15 days.

### *T. gondii* division and organelle observation

1 × 10^5^ tachyzoites were inoculated into HFFs and incubated at 37°C with 5% CO_2_ for 3 h waiting for an invasion, and divided into two groups of six wells each, with one group being treated with 100 μM Piceatannol and the other with DMSO as control. After 24 h, IFA was performed to observe the IMC, apicoplast and nuclei of the parasites by using rabbit anti-IMC1 antibody, mouse anti-ACP (acyl carrier protein) antibody and Hoechst dye, respectively.

### Cytotoxicity test

Cytotoxicity of piceatannol was evaluated in Vero cell lines using CCK-8 reagent (Beyotime, Shanghai, China). Vero cells (5,000 cells/well) were cultured in 96-well-plates at 37°C and 5% CO_2_ for 24 h. Sequentially diluted piceatannol from 20 μM to 500 μM was added to the host cells and incubated for 24 h, and then cell viability was determined using CCK-8 reagent according to the manufacturer's instructions. Absorbance was measured at 450 nm using a Microplate Absorbance Reader (BioRad, Hercules, CA, United States). DMSO was set as a control. Inhibition rate = (OD _DMSO_-OD _PIC_)/OD _DMSO_. The cytotoxicity experiment was performed in triplicate.

## Data availability statement

The original contributions presented in the study are included in the article/supplementary material, further inquiries can be directed to the corresponding author.

## Ethics statement

The animal study was reviewed and approved by Institutional Animal Care and Use Committee of Guangxi University.

## Author contributions

XS conceived and designed the study. YJ performed the experiments. YJ and XS analyzed the data and drafted the manuscript. DH helped in manuscript writing. YS helped in cytotoxicity test. All authors read and approved the final manuscript.

## Funding

This work was supported by the Specific Research Project of Guangxi for Research Base and Talents (Grant No. AD22035040), and the Natural Science Foundation of Guangxi Zhuang Autonomous Region (Grant No. 2021GXNSFBA220004).

## Conflict of interest

The authors declare that the research was conducted in the absence of any commercial or financial relationships that could be construed as a potential conflict of interest.

## Publisher's note

All claims expressed in this article are solely those of the authors and do not necessarily represent those of their affiliated organizations, or those of the publisher, the editors and the reviewers. Any product that may be evaluated in this article, or claim that may be made by its manufacturer, is not guaranteed or endorsed by the publisher.
